# A Lane-Changing Decision-Making Model of Bus Entering considering Bus Priority Based on GRU Neural Network

**DOI:** 10.1155/2022/4558946

**Published:** 2022-09-24

**Authors:** Wanjun Lv, Yongbo Lv, Jianwei Guo, Jihui Ma

**Affiliations:** School of Traffic and Transportation, Beijing Jiaotong University, Beijing 100044, China

## Abstract

A mandatory lane change occurs when buses are ready to enter the station, which will easily cause a reduction of urban road capacity and induce traffic congestion. Using deep learning methods to make lane-changing decisions has become one of the research hotspots in the field of public transportation, especially with the development of the Cooperative Vehicle-Infrastructure System. Aiming at the exploration of the bus lane-changing rules and decisions during entering, we built a GRU neural network model considering bus priority by using the first real-world V2X (vehicle to everything) dataset. Firstly, we illustrated the image and point cloud data processing by coordinate transformation. Secondly, the Kalman filtering algorithm was used to evaluate the vehicle state. Combined with the bus priority rules, we propose a flexible right-of-way lane in front of the bus stop. And then, we obtain the feature variables as inputs to the model. The XGBoost algorithm was chosen to train the GRU model. Results show that the model has higher identification accuracy for lane-changing maneuvers by comparison with other models. It plays a very important role in providing a decision basis for more refined bus operation management.

## 1. Introduction

Lane-changing behavior plays an important role in traffic flow theory. There are two types of lane-changing operations, namely, mandatory lane-changing (MLC) and discretionary lane-changing (DLC) [1]. Recently, researchers have shown an increased interest in the MLC maneuvers but paid less attention to the DLC scenes [2, 3]. MLC means that drivers are forced to leave the current lane because external factors have disturbed them or they are planning to reach their destination. A bus changing lanes and entering a station is one of the typical scenarios of MLC. Within the vicinity of the station, the bus must complete the lane change and prepare to stop at the station. Therefore, drivers are likely to adopt a radical lane change strategy, causing the surrounding vehicles to slow down, change lanes, and even queue, resulting in traffic congestion. More importantly, due to the large size of the bus itself, it will block the sight of the surrounding car drivers when changing lanes, which will easily lead to traffic accidents. Therefore, it is very necessary to study lane-changing behavior during the bus entering process.

One of the most significant current discussions in lane-changing is decision-making. Early lane change decision methods were mainly rule-based methods, which determined whether a vehicle met certain lane change rules by building a set of decision rules [4, 5]. Furthermore, models based on discrete choices are also used in lane change decisions [6–8]. This kind of model can take driver characteristics into account and at the same time has a simpler structure. However, the influence of different factors on the outcome of lane change is linear in these models, whereas lane change behavior is a process of coupled influence of multiple factors, and the relationship among the factors is often nonlinear. In recent years, in order to represent the nonlinear relationship between the input and output data more accurately, machine learning algorithms are increasingly being applied to lane-changing decision-making [9], such as support vector machine (SVM) [10], Bayesian filtering (BF) [11], and so on.

For MLC scenarios, many previous studies have focused on lane-changing behaviors in freeway on-ramp merging areas. There are fewer methods that have tried to solve the important issues of the lane-changing maneuvers that occur on buses entering. With the development of autonomous driving (AD) technology and the cooperative vehicle infrastructure system (CVIS), it is easier to collect enough bus-related data for machine learning algorithm training.

The purpose of this research is to model the bus lane-changing decision-making process using the first real-world V2X dataset for CVIS, with attention to the flexible bus priority along the bus entering route. The main contributions of this study are as follows:

1. This paper focuses on the process of bus lane-changing decision-making, one of the MLC behaviors, which can seriously affect traffic conditions and is rarely explored by previous research studies.
2. Based on real traffic data, a GRU neural network is used to solve the nonlinear bus lane-changing decision-making problem during entering. Combining the V2X technology, we take the bus priority into account, which is helpful to update real-time feature variables.

Section 2 reviews the literature on lane-changing methods.  Section 3 is concerned with the methodology used for this study. The training results and discussion are shown in Section 4. Finally, Section 5 gives a brief summary and areas for further research.

## 2. Related Work

Lane-changing is one of the common driving behaviors in traffic flow. Gipps [4] is the first scholar who built the vehicle lane-changing decision model to test the factors that affect drivers' lane-changing decisions in urban traffic. He proposed a logical frame for the lane-changing decision process. To better understand the mechanisms of the lane-changing model, other authors expanded the investigations by examining the diversified aspects of expressways and urban roads, including the lane-changing probability [12], drivers' aggressive and courtesy behaviors [13], and the triggered negative effects [14]. On the other hand, some authors also tried to develop Gipps's model. Kesting et al. first demonstrated the minimizing overall braking induced by Lane Change (MOBIL) model and considered the vehicle acceleration changes on the basis of the previous model [5]. As connected vehicles appeared, there was a large volume of published studies describing the role of cooperative merging strategies [15] and optimization algorithms to reveal the detailed mandatory lane-changing behaviors on freeways [16].

Besides, a considerable amount of literature uses utility theory to investigate lane-changing behaviors. The study of utility theory was first carried out by Ahmed et al. to examine the process of lane-changing decision-making on freeways. Toledo et al. integrated DLC and MLC behaviors by using the utility model [17]. Sun and Elefteriadou provided an in-depth analysis of the influences of drivers' characteristics on lane-changing behaviors by observing drivers' profiles and corresponding vehicle trajectory data [18, 19].

Recently, a number of machine learning-based lane-changing decision models have also emerged. Tang et al. utilized an adaptive learning approach to develop a fuzzy neural network-based prediction model for lane change behavior [20]. In response to the problem that it is difficult to model the multiparameter nonlinear features in the lane change decision process, the authors' team developed a support vector machine-based lane change decision model [21]. A neural network-based lane change decision model was developed in the paper [22]. Multilayer perceptron (MLP) and convolutional neural network (CNN) architectures were combined to demonstrate how the lane change acceptance of a specific driver can be learned from lane change intention and the surrounding environment. The work [23] proposed a deep reinforcement learning (DRL)-based motion planning strategy for autonomous driving tasks in highway scenarios where an autonomous vehicle merges into two-lane road traffic flow and realizes the lane-changing maneuvers.

These existing LC models were mostly developed for freeways and are not likely to be applied to buses that are entering. In the above analysis, we introduce the GRU neural network to improve the MLC decision process during bus entering.

## 3. Materials and Methods

### 3.1. Data Processing

The DAIR-V2X dataset is the first large-scale, multimodality, multiview dataset captured from real scenarios for CVIS and contains data captured from the Vehicle-Infrastructure Cooperative view [24]. It contains 71,254 LiDAR frames and 71,254 camera frames captured in intersection scenes where a well-equipped vehicle passes through intersections with infrastructure sensors deployed. 40% of the frames are captured from infrastructure sensors and 60% of the frames are captured from vehicle sensors. All of them are precisely labeled by expert annotators. The dataset covers 10 km of city roads, 10 km of highways, 28 intersections, and 38 km^2^ of driving regions with diverse weather and lighting variations. The subdatasets are presented in Table 1.

**Figure 1:**
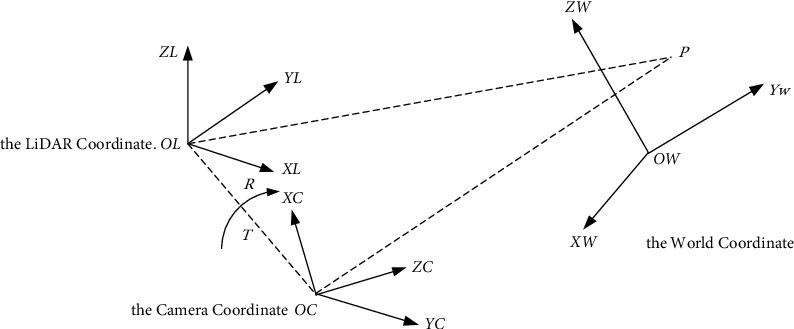
Schematic diagram of coordinate transformation.

In this paper, the DAIR-V2X–C dataset containing 38,845 frames of image data and 38845 frames of point cloud data were selected to train the model. In order to obtain information about the buses and their surrounding vehicles, the 3-dimensional (3D) LiDAR point cloud data and the 2-dimensional (2D) camera image set need to be correlated through spatial coordinate transformation. There are three coordinate systems used in the dataset, including the world coordinate, the camera coordinate, and the LiDAR coordinate. The schematic diagram of coordinate transformation is shown in Figure 1.

The Lidar-to-Camera transformation can be obtained by simply multiplying the Lidar-to-World and World-to-Camera transformations. In order to generate the depth map *D*_*G*_ of the ground plane with the same size as the image, we use the ground plane equation *G*(*α*, *β*, *γ*, *d*) and camera intrinsic *K*^3×3^ as shown in the following equation:

(1) $$\begin{array}{c} \left\{\begin{array}{c} {Z\left[x,y,1\right]}^{T}={K^{3\times 3}\left[X,Y,Z\right]}^{T}\\ G^{1\times 4}{\left[X,Y,Z,1\right]}^{T}=0 \end{array}\right.\text{,} \end{array}$$

where [*x*, *y*] is the pixel in the image coordinates and [*X*, *Y*, *Z*] is the corresponding 3D point in the camera coordinate that lies on the ground plane. Thus, the depth *Z* can be derived with the known 2D image points and the ground plane equation *G*.

After completing the matching of point cloud information and image information through coordinate conversion, we use the Kalman filtering algorithm to evaluate the vehicle state, including the 2 steps, namely, predicting and updating.

The first step is predicting, as shown in the following equation:

(2) $$\begin{array}{c} \left\{\begin{array}{c} x_{t+1}=A{\hat{x}}_{t}+Bu_{t}+w_{t}\\ P_{t+1}=A{\hat{P}}_{t}A^{T}+Q \end{array}\right.\text{,} \end{array}$$

where *x*_*t*+1_ is the predicted value of the system state vector at moment *t*+1. ${\hat{x}}_{t}$ and *u*_*t*_ are the optimal estimated value and input control vector of the state vector at moment *t*, respectively. Matrices *A* and *B* denote the state transfer matrix and control matrix, respectively. *w*_*t*_ is the noise of the system. *P*_*t*+1_ represents the predicted value of the state estimation covariance matrix at moment *t*+1. ${\hat{P}}_{t}$ means the optimal estimation of the state estimation covariance matrix at moment *t*. *Q* is the process noise covariance matrix.

The second step is updating, as shown in the following equation:

(3) $$\begin{array}{c} \left\{\begin{array}{c} K_{t+1}=P_{t+1}H^{T}{\left(HP_{t+1}H^{T}+R\right)}^{-1}\\ {\hat{x}}_{t+1}=x_{t+!}+K_{t+1}\left(z_{t+1}-Hx_{t+1}\right)\\ {\hat{P}}_{t+1}=\left(I-K_{t+1}H\right)P_{t+1} \end{array}\right.\text{,} \end{array}$$

where *K*_*t*+1_ denotes the Kalman gain coefficient. *H* is the measurement matrix. *R* is the measurement noise covariance matrix. ${\hat{x}}_{t+1}$ denotes the optimal estimate of the state vector after the update at moment *t*+1. *z*_*t*+1_ is the measurement value at moment *t*+1. ${\hat{P}}_{t+1}$ is the optimal estimation value of the state evaluation covariance matrix at moment *t*+1.

Because the bus lane-changing process does not involve external inputs to the system, both *B* and *w*_*t*_ in (2) are not taken into account. In this paper, we assume that the vehicle is treated as a uniform acceleration motion in each sampling period. Suppose the state vector is [*x*, *v*, *a*]^*T*^, which means position, speed, and acceleration. *A*_*k*_ is shown in (4). *H* is the unit matrix.

(4) $$\begin{array}{c} A_{k}=\left[\begin{array}{ccc} 1 & t & \frac{t^{2}}{2}\\ 0 & 1 & t\\ 0 & 0 & 1 \end{array}\right]\text{.} \end{array}$$

The state estimation of the target vehicle by applying the Kalman filtering algorithm jointly to the 3D LiDAR point cloud data and image data can obtain the position, velocity, and acceleration state information of the target vehicle.

### 3.2. Extraction of Feature Variables

**Figure 2:**
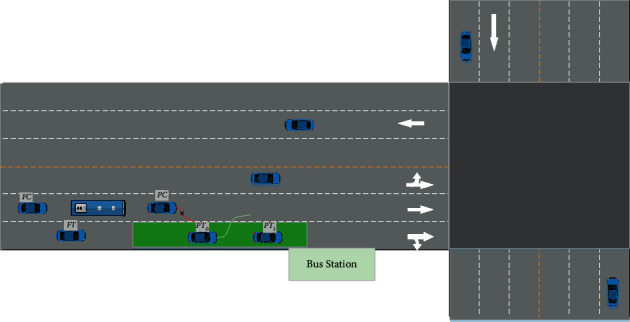
The bus priority of right-of-way when approaching the station.

Targeting buses' apparently purposeful and time-sensitive lane-changing behavior, it is necessary to guarantee the priority of right-of-way during their approach to the station. The process is illustrated in Figure 2.

The green area is the flexible right-of-way lane upstream of the bus stop, the length of which is determined by the traffic and roadway network status. *FT* is the upstream vehicle in the adjacent lane that needs to slow down or change lanes to avoid collision during the bus switches into this lane. *PT*_0_ is located downstream in the flexible right-of-way lane and will not turn right in the intersection, so it is requested to leave this lane; *PT*_1_ is located downstream in the flexible right-of-way lane and needs to turn right in the intersection, so it is not necessary to leave the current lane; *PC* is the vehicle located downstream in the lane where the bus is located. It is not allowed to change in the green area. The *FC* is the vehicle located upstream in the lane where the bus is located. Vehicles in other lanes do not directly affect the bus and are not analyzed.

According to Gipps' safe distance rules [4], *D*_*PC*_ and *D*_*PT*_ indicate the safe distance between the subject vehicle (the bus) and the preceding vehicle in the current lane and the target lane, respectively.

(5) $$\begin{array}{c} \left\{\begin{array}{c} D_{PC}^{i}=v_{b}^{i}\tau +\frac{{\left(v_{b}^{i}\right)}^{2}}{2d_{b}}-\frac{{\left(v_{PC}^{i}\right)}^{2}}{2d_{PC}}\\ D_{PT}^{i}=v_{b}^{i}\tau +\frac{{\left(v_{b}^{i}\right)}^{2}}{2d_{b}}-\frac{{\left(v_{PT}^{i}\right)}^{2}}{2d_{PT}} \end{array}\right.\text{,} \end{array}$$

where *τ* indicates the reaction time of bus driver. *v*_*b*_^*i*^, *v*_*PC*_^*i*^, and *v*_*PT*_^*i*^ are the initial velocity of the bus, PC, and PT in each sampling period, respectively. *d*_*b*_,*d*_*PC*_ , an d *d*_*PT*_ are the maximum deceleration of the corresponding vehicle.

Similarly, the safe distance between the bus and the following vehicle before and after the bus changes the lane (enters the green area in Figure 2) can be formulated as follows:

(6) $$\begin{array}{c} \left\{\begin{array}{c} D_{FC}^{i}=v_{FC}^{i}\tau +\frac{{\left(v_{FC}^{i}\right)}^{2}}{2d_{FC}}-\frac{{\left(v_{b}^{i}\right)}^{2}}{2d_{b}}\\ D_{FT}^{i}=v_{FT}^{i}\tau +\frac{{\left(v_{FT}^{i}\right)}^{2}}{2d_{FT}}-\frac{{\left(v_{b}^{i}\right)}^{2}}{2d_{b}} \end{array}\right.\text{,} \end{array}$$

where *v*_*FC*_^*i*^ and *v*_*FT*_^*i*^ denote the initial velocity of the FC and FT in each sampling period, respectively. *d*_*FC*_ and *d*_*FT*_ represent the maximum deceleration of the corresponding vehicle.

In a real-world lane-changing scenario, the bus tends to finish the lane-changing process as soon as possible. Some studies adopt the time ratio *t*_*b*_^*f*^/*t*_max_ to represent this characteristic, where *t*_*b*_^*f*^ denotes the time used to finish the lane-changing process and *t*_max_ is the longest allowable time for lane-changing maneuver. The traffic efficiency description in this paper is developed by the above-mentioned method, and the index can be calculated as follows:

(7) $$\begin{array}{c} \frac{t_{b}^{f}}{t_{\max}}=\frac{\sum_{i}^{N}x_{b}^{if}-x_{b}^{i0}/v_{b}^{i}}{t_{\max}}\text{,} \end{array}$$

where *x*_*b*_^*if*^ and *x*_*b*_^*i*0^ represent the final and initial position of the *i*th sampling period, respectively. *t*_*b*_^*f*^ can be approximately calculated by ∑_*i*_^*N*^*x*_*b*_^*if*^ − *x*_*b*_^*i*0^/*v*_*b*_^*i*^.

After considering the safety and efficiency, we can obtain the feature variables as shown in Table 2.

Thanks to the advantages of CVIS, using V2X communication seems to be the logical solution. Other vehicles on the road can check whether there are approaching buses from behind periodically within a specific sensing distance. These feature parameters can be transmitted by vehicle-to-vehicle technology according to the expected wireless communication range of V2X systems. In case a vehicle detects an approaching bus that is entering the station in its adjacent or current lane, the vehicle will determine whether it affects the ideal bus operation to guarantee its priority. Of course, this paper assumes that all vehicles have real-time communication capabilities and does not reject the bus lane-changing requirement.

### 3.3. Lane-Changing Decision-Making Model

**Figure 3:**
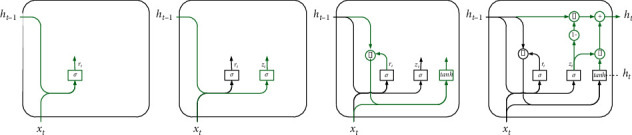
GRU structure and calculation process. (a) Step 1 of GRU. (b) Step 2 of GRU. (c) Step 3 of GRU. (d) Step 4 of GRU.

The gated recurrent unit (GRU) network is one of the variants of long-short-term memory (LSTM) networks in recurrent neural network (RNN), which mainly replaces the two gating units of LSTM (forgetting gate and input gate) with one gating unit (update gate). The GRU model is more streamlined than the standard LSTM model, with only two gating units in the hidden layer neuron structure and fewer network parameters, and its model structure is shown in Figure 3. It has been well developed and applied in the field of time series prediction.

The GRU neural network works similarly to the LSTM in which it has two gates, namely, a reset gate and an update gate. Both the reset gate and the update gate receive the hidden state *h*_*t*−1_ at the previous moment and the input information *x*_*t*_ at the current moment and the output gating signals are denoted as *r*_*t*_ and *z*_*t*_, respectively. The gating unit also consists of a sigmoid function and a dot product operation. The GRU works as follows.

Short time series dependencies are obtained via the reset gate as follows:

(8) $$\begin{array}{c} r_{t}=\sigma \left(W_{xr}x_{t}+W_{hr}h_{t-1}+b_{r}\right)\text{.} \end{array}$$

Calculate update gate as follows:

(9) $$\begin{array}{c} z_{t}=\sigma \left(W_{xz}x_{t}+W_{hz}h_{t-1}+b_{z}\right)\text{.} \end{array}$$

The candidate vector is obtained after updating as follows:

(10) $$\begin{array}{c} {\tilde{h}}_{t}=\tanh\;\left(W_{x\tilde{h}}x_{t}+W_{h\tilde{h}}\left({r_{t}\;h}_{t-1}\right)+b_{c}\right)\text{.} \end{array}$$

Update memory to get hidden layer output results as follows:

(11) $$\begin{array}{c} h_{t}=\left(1-z_{t}\right)\;h_{t-1}+z_{t}\;{\tilde{h}}_{t}\text{,} \end{array}$$

where *σ* is the sigmoid function and tanh is the tanh function. *W*_*xz*_,*W*_*xr*_, and *W*_*xc*_ are the weight matrices from *x*_*t*_ to the update gate, reset gate, and candidate hidden states, respectively; *W*_*hz*_,*W*_*hr*_, and $W_{h\tilde{h}}$ are the weight matrices from *h*_*t*−1_ to the update gate, reset gate, and candidate hidden states, respectively; *b*_*z*_, *b*_*r*_, and *b*_*c*_ are the bias.

**Figure 4:**
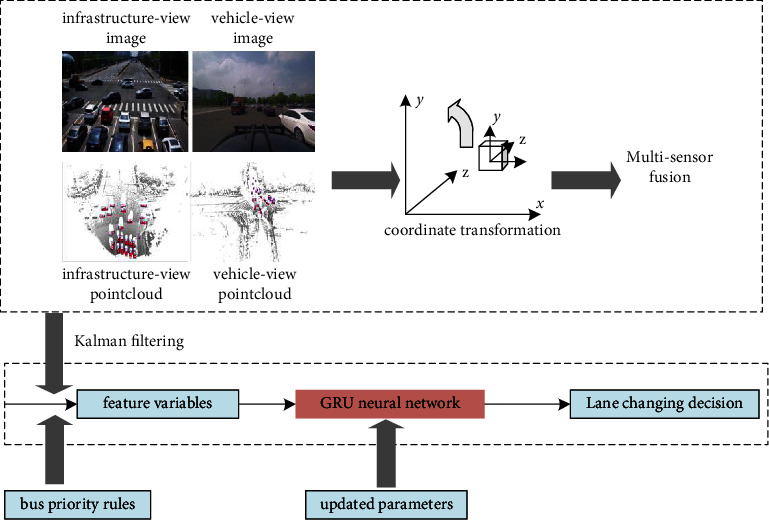
The lane-changing decision-making model of bus entering.

The lane-changing decision-making model of the bus entering is illustrated in Figure 4. Firstly, image and point cloud data are matched by coordinate transformation. Then, using the Kalman filtering method and considering the bus priority rules during the entering process, we can obtain the state of corresponding vehicles and extract feature variables, namely, the inputs of the GRU neural network.

## 4. Results and Discussion

### 4.1. Testing Data and Evaluation Metrics

**Figure 5:**
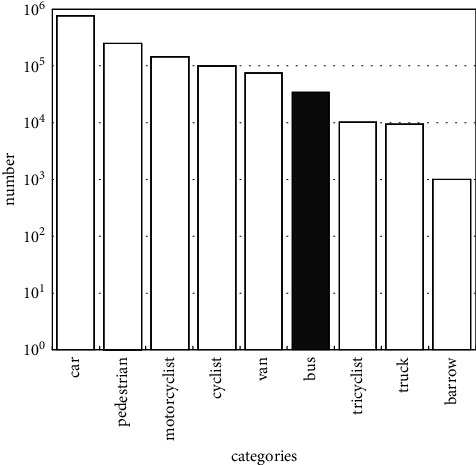
The number of different vehicles categories in DAIR-V2X dataset.

We adopted the first large-scale DAIR-V2X dataset, which provides high-quality traffic data for research. The captured images obtained by cameras and the point clouds scanned by the LiDAR are calibrated and aligned between different sensors. The transformations among the world, the camera, and LiDAR are obtained, as well as the ground plane equation. The 2D-3D joint annotation is carried out by projecting the point clouds onto the images and adjusting the 3D bounding boxes manually to fit the 2D instance. As shown in Figure 5, there are a total of 9 kinds of traffic elements in the dataset. The objective of this paper is to show that, the corresponding number of samples is more than 60,000.

We used the scaled exponential linear unit (SeLU) as the activation function in the hidden layers. Compared with the traditional sigmoid and the popular rectified linear unit (ReLU), SeLU can converge towards zero mean *y* and unit variance automatically. Therefore, it has the same effect as batch normalization, which means that exploding and vanishing gradients can be avoided. The SeLU activation function is shown as follows:

(12) $$\begin{array}{c} SeLU\left(x\right)=\lambda \left\{\begin{array}{c} x\;if\;x>0\\ \alpha ∙e^{x}-\alpha \;if\;x\leq 0 \end{array}\right.\text{,} \end{array}$$

where *λ* and *α* are two parameters with typical values, namely, 1.0507 and around 1.6733, respectively.

According to our previous research [25], the operating time of the XGBoost algorithm is much faster than that of other programs on a single device. It can scale up to billions of examples in the case of distributed or limited memory. We chose XGBoost algorithm to train the GRU neural network in this paper.

An appropriate evaluation framework can help to estimate performance. Basically, samples in classification problems are divided into four categories, including true positives, true negatives, false positives, and false negatives. We introduce this evaluation framework for the model in Table 3.

TP and FN are the probabilities of actual lane-changing events correctly and wrongly classified as lane-changing and lane keeping events, respectively. FP and TN are the numbers of actual lane keeping events wrongly and correctly classified as lane-changing and lane keeping events, respectively. The following evaluation indicators (13) and (14) calculated by these four parameters are chosen in this paper:

(13) $$\begin{array}{c} accuracy\left(ACC\right)=\frac{TP+TN}{TP+TN+FP+FN}\text{,} \end{array}$$

(14) $$\begin{array}{c} true\;positive\;rate\left(TPR\right)=\frac{TP}{TP+FN}\text{.} \end{array}$$

Furthermore, the ROC curve can describe the relationship between TP and FP. The ROC score which calculates the area under the ROC curve is also a judging basis for the effect of algorithm.

### 4.2. The Optimal GRU Structure

In the paper [26], it was stated that overfitting in training neural networks was serious and should be given more attention. When overfitting happens, the error on the testing dataset is still high although the error of the model on the training dataset could be ignored. To avoid this phenomenon, various network structures, from simple to complicated, are tested in this paper. The different neural network structures are illustrated in Table 4 (where 0 means that there is no layer in the corresponding structure).

**Figure 6:**
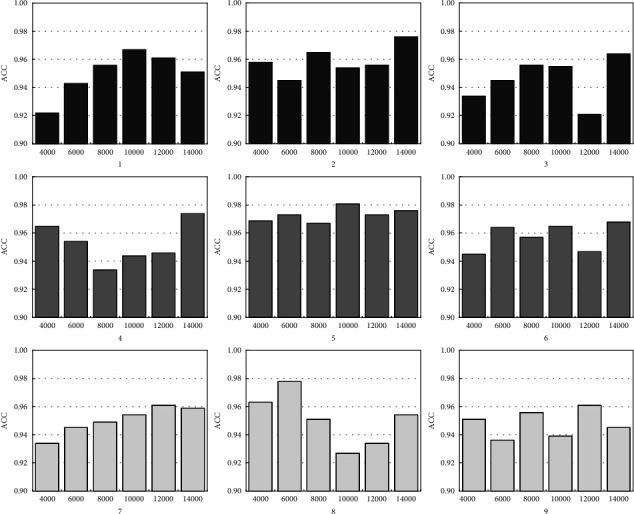
ACC values of different GRU network structures.

We use the cross-validation method to avoid overfitting, and 30% of the training samples are chosen as the validation set. Further statistical tests revealed that the model works efficiently with the help of the cross-validation method. We separate all the samples into 3 parts: 30% as the training dataset, 30% as the validation set, and the remaining 40% as the test dataset. For all the neural network schemes in Table 4, combined with the flexible right-of-way lane in front of the bus stop, generally at least 4,000 to 14,000 samples (10% to 40% of the total sample) are required. The results are shown in Figure 6.

**Figure 7:**
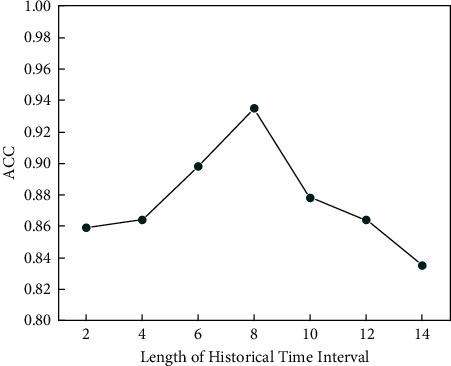
ACC values of different length of the historical time interval.

Results show that structure 5 (3 hidden layers that contain 30, 10, and 0 neurons, respectively), trained by 10,000 samples, obtained the best performance. Furthermore, we selected 10,000 training samples to test different lengths of the historical time interval. Results show that considering the 8-second historical input is always the best choice.  Figure 7 gives an example of ACC of the model.

To examine the accuracy of the bus lane-changing model in this paper, we compared the performance of the stochastic gradient descent (SGD) algorithm, the Random Forest model, and the SVM model with our model in the dataset. The indexes of different models are presented in Table 5. It can be seen that the GRU model in this paper performs better than other models. The ACC value of our model is up to 92.45%, much higher than that of others. It is worth noting that the TPR can guarantee the safety during bus lane-changing, which is vital for the driver to operate the lane-changing successfully. Hence, it could conceivably be noticed that the higher the value of TPR is, the safer the lane-changing will be. At the same time, the false negative would not have a negative impact on driving safety because it only leads to the loss of an opportunity to change lanes. Therefore, it is necessary to pay more attention to the accuracy of lane-changing maneuvers. The results of the model indicate that it is a safe and effective bus lane-changing decision-making system.

**Figure 8:**
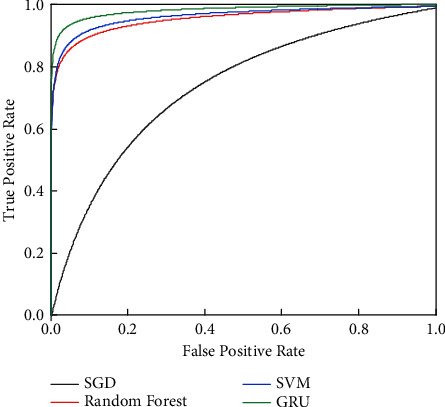
Comparison of ROC curves among different models.

Figure 8 compares ROC curves among SGD, Random Forest, SVM, and GRU. It is apparent that the performance of the proposed GRU neural network is better than that of other binary classifiers, which means that the same conclusion can be drawn as what we get in Table 5. The gated branch plays an important role in correlation analysis, which explicitly extracts the characteristics of the feature variables, which is helpful to the lane-changing decision.

## 5. Conclusion

Based on the DAIR-V2X dataset, this study builds a lane-changing decision-making model of a bus entering based on the GRU neural network. Image and point cloud data are matched by coordinate transformation to obtain the number of bus vehicles. To reduce the noise interference, the Kalman filtering algorithm is used to evaluate the buses' position, speed, and acceleration. Meanwhile, the proposed method takes the bus priority rules into consideration when it enters the station, thus getting novel feature variables as input vectors. After calculating the experimental data, we choose the network structure “3 hidden layers that contain 30, 10, and 0 neurons, respectively,” as the optimal stricture and 8 seconds as the length of the historical time interval because the outcome performs best. By comparison and verification, it was proved that the proposed model has higher accuracy. The ACC value of our model is up to 92.45%.

Further research regarding the role of lane-changing would be worthwhile. This study considers the rules to ensure the priority of buses entering with the precondition that there is a 100% penetration rate of V2X technology in all vehicles. In a realistic road environment, the existence of disobedience to the lane-changing rules needs to be considered.

## Figures and Tables

**Table 1:** The details of DAIR-V2X dataset.

DAIR-V2X dataset	View	Image	Point cloud
DAIR-V2X–C	Vehicle-infrastructure cooperative	38845	38845
DAIR-V2X–V	Single vehicle	22325	22325
DAIR-V2X–I	Infrastructure	10084	10084

**Table 2:** Bus entering lane change decision feature variables.

Feature variable	Meaning
*v*_*b*_	The velocity of the bus
*x*_*b*_	The position of the bus
*d*_*b*_	The maximum deceleration of the bus
*v*_*n*_, *n* ∈ {*PC*, *PT*, *FC*, *FT*}	The velocity of *PC*, *PT*, *FC*, *FT*
*x*_*n*_, *n* ∈ {*PC*, *PT*, *FC*, *FT*}	The position of *PC*, *PT*, *FC*, *FT*
*D*_*n*_, *n* ∈ {*PC*, *PT*, *FC*, *FT*}	The safe distance between the bus and *PC*, *PT*, *FC*, *FT*
*d*_*n*_, *n* ∈ {*PC*, *PT*, *FC*, *FT*}	The maximum deceleration of *PC*, *PT*, *FC*, *FT*
Τ	The reaction time of bus driver
*t*_*b*_^*f*^	The time used to finish the lane-changing process
*t*_max_	The longest allowable time for lane-changing maneuver

**Table 3:** Evaluation framework of bus lane-changing decision model.

		Training condition	
		Bus lane changing	Bus lane keeping
True condition	Bus lane changing	TP	FN
	Bus lane keeping	FP	TN

**Table 4:** GRU neural network tested.

Structure	Hidden layer		
	1	2	3
1	30	0	0
2	50	0	0
3	100	0	0
4	10	10	0
5	30	10	0
6	50	10	0
7	10	10	5
8	10	10	10
9	30	10	10

**Table 5:** Evaluation values of different models for lane-changing.

Model	ACC (%)	TPR (%)
SGD	76.34	95.41
Random forest	81.23	93.25
SVM	89.35	90.33
GRU	92.45	96.68

## Data Availability

The labeled dataset used to support the findings of this study is available from the corresponding author upon request.
